# Home-Based Intermittent Pneumatic Compression Therapy: The Impact in Chronic Leg Lymphedema in Patients Treated for Gynecologic Cancer

**DOI:** 10.3390/healthcare10040638

**Published:** 2022-03-28

**Authors:** Yoon Kim, Seonghee Kim, Ji Young Lim, Chea Min Hwang, Myoung-Hwan Ko, Ji Hye Hwang

**Affiliations:** 1 Department of Physical and Rehabilitation Medicine, Samsung Medical Center, School of Medicine, Sungkyunkwan University, Seoul 06351, Korea; cil3636@gmail.com; 2 Department of Rehabilitation Medicine, National Rehabilitation Center, Seoul 01022, Korea; 3 Research Institute for Future Medicine, Samsung Medical Center, Seoul 06351, Korea; a2387952@naver.com (S.K.); ktshgh2@naver.com (C.M.H.); 4 Department of Physical Therapy, Graduate School of Medical Sciences, Konyang University, Daejeon 35365, Korea; deartojay@gmail.com; 5 Department of Physical Medicine and Rehabilitation, Jeonbuk National University Medical School, Jeonju 54907, Korea; mhko@jbnu.ac.kr

**Keywords:** intermittent pneumatic compression (IPC), lymphedema, home therapy, quality of life, self-management

## Abstract

We conducted a prospective study of cancer patients to investigate the efficacy, quality of life, satisfaction, and safety of a home-based intermittent pneumatic compression (IPC) device during the maintenance phase of lower extremity lymphedema. This device has a unique mode designed to mimic the manual lymphatic drainage (MLD) technique and thereby gently facilitate lymphatic draining of proximal extremities. Thirty patients with stage 3 chronic secondary unilateral leg lymphedema in the maintenance phase underwent IPC and conventional compression therapy for 4 weeks at home. The participants were guided to use 1 h course (30 min of MLD-mimicking mode and 30 min of conventional mode) of IPC device twice a day for 4 weeks. We assessed the patients’ limb-volume measurement, quality of life (QOL), and satisfaction four times. There were no significant time-dependent interactions in the inter-limb volume difference ratio (V*_ratio_*). In a subgroup analysis, participants who used the home-based IPC device and maintained their routine self-maintenance program of short-stretch bandages (group B, *n* = 21) showed a more significant decline in V*_ratio_* than those who did not maintained their routine care (group A, *n* = 9). All scores of QOL decreased significantly after the intervention without subgroup difference. All participants were satisfied with the 4-week intervention. This study demonstrated that a home-based IPC device with an MLD-mimicking program is a useful option for maintaining the volume of limbs and improving the QOL of patients with stage 3 chronic leg lymphedema during the maintenance phase.

## 1. Introduction

Lymphedema is an abnormal extracellular accumulation of interstitial fluid containing proteins, cytokines, extravascular blood cells, and products of parenchymal and stromal cells [1,2]. It occurs as a result of an insufficient lymphatic system or decreased lymphatic transport, which can be caused by inflammation, trauma, surgery, or irradiation [3]. Chronic impairment of the lymphatic system produces secondary changes in the soft tissue, resulting in fibroblast, keratinocyte, and adipocyte proliferation, eventually destroying the elastin fibers of the skin [4,5].

In gynecologic cancer patients who undergo surgery, the most devastating chronic complication is leg lymphedema. In addition to cosmetic changes in the affected limb, patients with lymphedema experience various chronic complications such as recurrent soft tissue inflammation, lymphorrhea, and functional impairment of the affected limb [6]. Patients can also experience diverse forms of psychological and social distress [7].

Unfortunately, lymphedema has no definite cure [8]. Currently used therapeutic techniques focus on minimizing the volume and complications and maintaining or restoring the function of the affected limb [9]. The American Cancer Society recommends complex decongestive therapy (CDT), a combination of skin care, manual lymphatic drainage (MLD) massage, multilayer compressive bandaging, compressive garments, and intermittent pneumatic compression (IPC) [10]. CDT has been shown to produce an immediate volume reduction in patients with leg lymphedema. However, edema may gradually increase within six months of CDT, with edema of the proximal part increasing more than that of the distal part [11].

Patients with chronic lymphedema receive two phases of treatment: decongestive and maintenance. During the decongestive phase, patients undergo MLD from a specialized physical therapist and compression therapy using short-stretch compression bandages. During the maintenance phase, patients spend a tremendous amount of time and effort attempting to maintain a reduced edematous leg volume during the maintenance phase. Additional therapeutic exercises and skin care are provided. Subsequently, patients in the maintenance phase attempt to maintain the reduced limb volume they achieved through the intensive treatment of the decongestive phase. In the maintenance phase, patients are instructed to wear a gradient pressure garment during the day and compression bandages at night, and to continue their skin care and therapeutic exercise routines [12].

In 2018, a randomized controlled trial concluded that 18 days of multilayer bandaging followed by the use of an elastic garment for 24 weeks significantly reduced the volume of the affected limb, and that the reduction lasted longer than that of garment-only treatment [13]. Various compression garments have been tested to relieve the burden of daily bandaging, and they generally improve patient compliance. The International Society of Lymphology (ISL) reported that patient self-care and risk reduction strategies could support the maintenance of edema reduction. In a consensus document report by the ISL, IPC was introduced as a supportive measure for patients who could not complete both phases of CDT [14].

A home-based IPC device may be an option for patients with chronic lymphedema during the maintenance phase. Since the introduction of IPC therapy with a non-gradient single-chamber pressure cuff for the treatment of lymphedema in the early 1950s [15], therapeutic outcomes have gradually improved through the use of multi-chamber pressure cuffs and sequentially segmented pressure gradients from the distal to the proximal part of the limb [16,17]. A recent innovation in IPC devices is an MLD-mimicking program, which is expected to effectively clear preexisting lymphatic fluids from the proximal trunk and proximal limb. Only one previous study tested this new-generation IPC device for the treatment of lymphedema [18].

The primary aim of this prospective interventional study was to evaluate the efficacy of a home-based IPC device with a MLD-mimicking program for the treatment of gynecological cancer patients with secondary unilateral leg lymphedema during the maintenance phase. In this single-arm study, gynecologic cancer patients with secondary unilateral leg lymphedema were given a therapeutic intervention and then followed over time to observe their response to the inter-limb volume difference ratio, quality of life (QOL) over time, and device satisfaction.

## 2. Materials and Methods

### 2.1. Participants

A prior sample size calculation was performed on the basis of a previous study on the long-term therapeutic effect of IPC in lymphedema patients [1]. Using G*Power software version 3.1.9.2 (Dūsseldorf, Germany), 25 subjects were required to detect an effect size of f = 0.5862 at a significance level of 0.05 and a power of 0.80. We recruited 30 participants to account for a dropout rate of 20%.

Patients diagnosed with chronic unilateral secondary leg lymphedema after gynecological cancer treatment in the lymphedema outpatient clinic of a tertiary hospital between March 2019 and June 2019 were enrolled in this study. The inclusion criteria were as follows: patients aged 20–70 years who diagnosed stage 3 chronic unilateral secondary leg lymphedema; patients who had undergone one to two cycles of decongestive phase treatment; patients whose limb volume change has been stable within 10% for the past 3 months and a current inter-limb volume difference of more than 10%; patients who had capacity for self-maintenance care for lymphedema (multilayer limb bandaging, compression garments, and MLD). To classify the stage of lymphedema in each patient, a lymphedema clinic specialist diagnosed the disease stage according to the ISL criteria [19]. In addition, on their first visit to the clinic, all patients were educated in self-maintenance care including exercise and self-bandaging by a physiotherapist with over 20 years of experience in lymphedema. With regards to subject selection, the physiotherapist evaluated the patients’ capacity for self-maintenance care.

Patients with bilateral leg lymphedema, current cancer metastasis, ongoing chemotherapy or radiation therapy, acute inflammation, venous thrombosis, or chronic venous insufficiency were excluded from this study. Patients with systemic etiologies of edema, such as congestive heart failure, and those taking medication that influenced body fluids or electrolytes were also excluded.

### 2.2. Study Design

This prospective, longitudinal, interventional study aimed to investigate whether the objective limb volume difference or subjective symptoms of lymphedema in patients with chronic secondary leg lymphedema in the maintenance phase changed after applying home-based IPC and conventional compression therapy. Demographic characteristics were assessed before the application of IPC and relevant medical data were collected from medical records. The primary outcome measure was the volume difference between the lower limbs, which was measured four times: before IPC (T0), after four weeks of IPC application (T1), and at one month (T2) and two months (T3) post-intervention. All participants provided written informed consent and the study was approved by the institutional review board of our institute (approval no. SMC2018-11-144).

### 2.3. Intervention

We used a device produced by Maxstar Corp. (Gimpo, Korea) that specializes in pneumatic compression appliance manufacturing. The IPC device (UAM-9306NB) was approved by the National Institute of Medical Device Safety Information (NIDS) of Korea (approval number: 18-4745). The device consists of a six-chamber pneumatic sleeve and a gradient-sequential pneumatic pump. Two programmed modes were used during the intervention. The first mode, which mimics the MLD massage technique, begins with the inflation of the proximal chamber. After reaching the target pressure, the next chamber inflates consecutively, whereas the initially inflated proximal chamber deflates. The inflation time of each chamber was 3 s, with a holding time of 1 s. The deflation and resting times of each chamber were 7 s (pressure setting: 40–60 mmHg). After 30 min of the first mode, the second mode, which is the conventional mode of sequential inflation from the distal to proximal chambers while sustaining the pressure of the previously inflated chambers, was applied for 30 min (pressure setting: 80–100 mmHg, inflation time of each chamber: 6 s, holding time: 1 s, deflation time of each chamber: 7 s). Participants were instructed to use the IPC device on a 1-h cycle twice a day for four weeks. Diaries were provided to check the duration of use and complications. Participants were instructed to maintain their routine self-maintenance care program for lymphedema (compression bandaging, compression garments, and MLD) during the intervention period. At the one-month post-intervention visit, patients were asked about self-care routine adherence (yes or no). If they answered that they did not comply, the examiner recorded the level of care that was not taken.

### 2.4. Outcome Measures

#### 2.4.1. Inter-Limb Volume Difference Ratio

An optoelectronic scanner (Perometer 350 S, Pero-System Messgeräte GmbH, Wuppertal, Germany), which showed excellent reproducibility in lymphedema patients [20], was used to measure the inter-limb volume differences of the participants. The participants were asked to sit in a seat without a backrest parallel to the rails moving along the measuring frame. The subject’s heel was placed on a footrest for support and the knee was positioned at 0°. The measuring frame was set up to move at a constant rate, performing a new measurement every 4.7 mm. All scans were stored in a database and automatically analyzed using specialized software provided by the manufacturer. In our study, we measured three leg volumes: the distal leg volume (distal volume: from the smallest circumference at the ankle [B-measure] to the calf circumference immediately below the knee bend [D-measure]), the whole leg volume (limb volume without the foot up to the mid-thigh region [F-measure]), and proximal leg volume (subtraction of the distal leg volume from the whole leg volume) [21].

The inter-limb volume difference ratio (V*_ratio_*) was calculated using the following formula:

$$V_{ratio}=\frac{V_{affected}-V_{unaffected}}{V_{unaffected}}\times 100\;\left(\%\right)$$

where V*_affected_* is the volume measured in the affected limb and V*_unaffected_* is the volume measured in the unaffected limb.

#### 2.4.2. Quality of Life

At every time point, the participants completed a QOL questionnaire. Our team modified and translated the lymphedema functioning, disability, and health questionnaire for lower limb lymphedema (Lymph-ICF-LL) to better suit the Korean culture. Similar to the original Lymph-ICF-LL, the modified questionnaire contained 28 questions divided into five domains: physical function, mental function, general tasks/household activities, mobility activities, and life domains/social life. Each question was scored from 0 (not at all) to 10 (a lot), with lower scores representing better QOL [22]. Participants completed each questionnaire based on their average experience of leg lymphedema in the preceding two weeks. The total scores and the five domain scores were calculated by summation.

#### 2.4.3. Participant Satisfaction

We gathered satisfaction questionnaires from all participants who completed the four-week intervention. The questionnaire contained six items related to the subjective effects of the IPC device on volume reduction, pain, heaviness, skin hardness, ease of use, and overall satisfaction with the home therapy offered by the device. Each question was answered using a 5-point scale from 1 (not at all) to 5 (very much).

Participants were excluded from the study for a significant adverse event related to the study intervention, including the presence of significant pain, tightness, stiffness, heaviness, or signs of inflammation.

### 2.5. Statistical Analysis

All statistical analyses were performed using SPSS, version 22.0 (IBM Corp, Armonk, NY, USA), and statistical significance was set at *p* < 0.05. Demographic and clinical characteristics are presented as mean ± standard deviation for continuous variables and as percentages for categorical variables. All continuous data were initially checked for normality using the Shapiro-Wilk test. Repeated measures of the V*_ratio_* of the proximal, distal, and whole leg volumes were analyzed using the Friedman test. The Friedman test and repeated-measures analysis of variance were used to evaluate time-to-time differences in the lymph ICF-LL. If there was a significant difference, the paired *t*-test and Wilcoxon signed-rank test were used for pairwise comparison.

For the subgroup analysis, based on compliance with compression bandaging, we divided the participants into two groups. Nine participants (group A) reported that they did not perform compression bandaging as usual during the intervention period, although they reported using the home IPC device as instructed in the study protocol. The remaining participants (group B, *n* = 21) reported that they properly used the home-IPC device and maintained their routine self-care maintenance program. Additional analyses using a two-sample *t*-test and Mann–Whitney U test were performed to compare the two subgroups. Comparison of V*_ratio_* and the lymph ICF-LL between two groups over times was carried out by using a Bonferroni corrected *p* value of 0.008 (0.05/6). Significant difference between two groups in satisfaction of IPC device intervention was defined as *p* < 0.05.

## 3. Results

### 3.1. General Characteristics

Thirty patients were enrolled in this study and completed the follow-up period. The demographic and clinical characteristics of the patients are presented in Table 1. The mean age of the participants was 56.57 ± 11.33 years, and all participants were female. The mean onset of lymphedema was 3.18 ± 2.58 years after cancer surgery, and the mean duration of lymphedema was 15.46 ± 7.57 years. The initial mean body mass index (BMI) of the participants was 23.87 ± 3.33, which is in the overweight range according to the World Health Organization Asian BMI classification [23]. More than half of the participants had been diagnosed with cervical cancer. All participants underwent cancer surgery with pelvic lymph node dissection, and 11 patients (36.7%) underwent chemotherapy or radiotherapy. The demographic and clinical characteristics of the subgroups (groups A and B) were similar (*p* > 0.05).

### 3.2. Time-Dependent Changes in Inter-Limb Volume Difference Ratio

**Figure 1:**
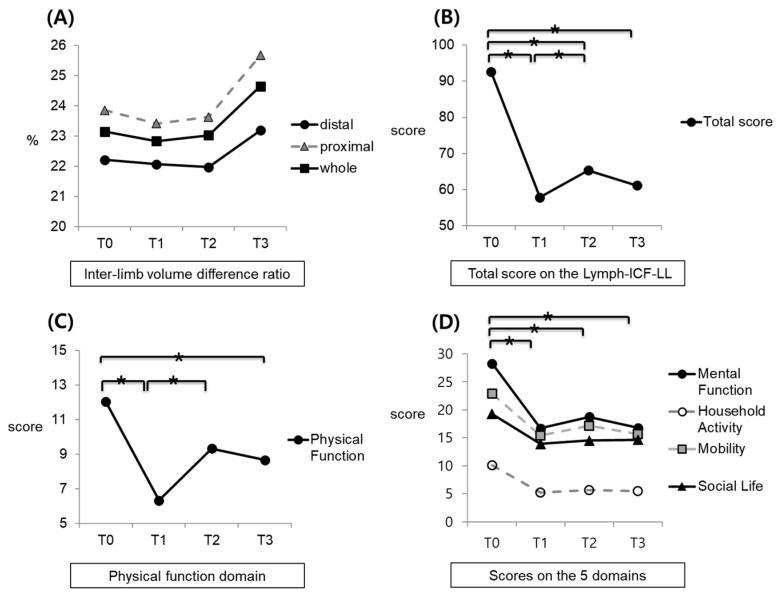
Impact of a home-based IPC device on inter-limb volume difference ratio, total ICF score, score on the physical function domain, scores on the four domains: (**A**) inter-limb volume difference ratio; (**B**) total score on the Lymph-ICF-LL, (**C**) scores on the physical function domain, and (**D**) scores on the 5 domains (mental function, household activity, mobility, and social life). T0: baseline; T1: post-intervention; T2: one-month follow-up; and T3: two-month follow-up. * *p* < 0.05.

No significant time-dependent effect was observed with regards to the V*_ratio_* (Table 2). Specifically, the distal limb V*_ratio_* showed a declining trend after the four-week intervention compared to baseline. This declining trend continued after one month of conventional care without the use of a home-based IPC device. At the two-month follow-up, the distal limb V*_ratio_* increased insignificantly compared to all previous time points. In the proximal limb and whole limb data, the V*_ratio_* decreased insignificantly after the four-week intervention period. However, this trend did not last until the one-month follow-up period. Changes in the V*_ratio_* across the four time points are shown in Figure 1A.

### 3.3. Time-Dependent Changes in the Inter-Limb Volume Difference Ratio between the Subgroups

We compared time-dependent V*_ratio_* changes based on participants’ self-reported compliance with their prescribed self-maintenance care for lymphedema during the intervention period. The participants showed different levels of compliance with self-maintenance care in only one category: multilayer compression bandaging with short-stretch bandages.

As shown in Table 3, group B, that properly used the home-IPC device and maintained their routine self-care maintenance program, presented a significantly larger V*_ratio_* decline than group A in the proximal, distal, and whole-limb measurements after the four-week intervention. At the one-month follow-up test, the distal limb V*_ratio_* in group B increased significantly compared to the post-intervention result, while a decrease was noted in group A. No other significant intergroup differences were seen between the data from the post-one-month and post-two-month tests.

### 3.4. Time-Dependent Changes in the ICF Domain Scores

The total ICF score and all ICF domain scores showed significant time-dependent changes at the four time points (Table 4, Figure 1B–D). The total ICF score decreased significantly after the four-week intervention compared to that at baseline (*p* < 0.001). Although the total score increased with slight significance after one month of conventional care without additional home-based IPC device usage, compared with the post-intervention period (*p* = 0.048), the follow-up scores at one and two months were still significantly lower than baseline (*p* < 0.001 and *p* < 0.001, respectively). Specifically, the physical function scores decreased significantly after the intervention compared to baseline (*p* = 0.001), and then increased significantly at the one-month follow-up compared with the post-intervention results (*p* = 0.009). At the two-month follow-up, the physical function scores decreased slightly compared to the one-month follow-up results, but these scores were still significantly lower than the baseline scores (*p* < 0.001).

The other four domains of the ICF (mental function, household activity, mobility, and social life) presented the same time-dependent significance: they all declined significantly from baseline to after the intervention, increased slightly at the one-month follow-up, and decreased slightly at the one-month follow-up compared to the previous time point. Even so, the one-month and two-month follow-up scores were significantly lower than the baseline scores.

### 3.5. Time-Dependent Changes in the ICF Domain Scores between the Subgroups

**Figure 2:**
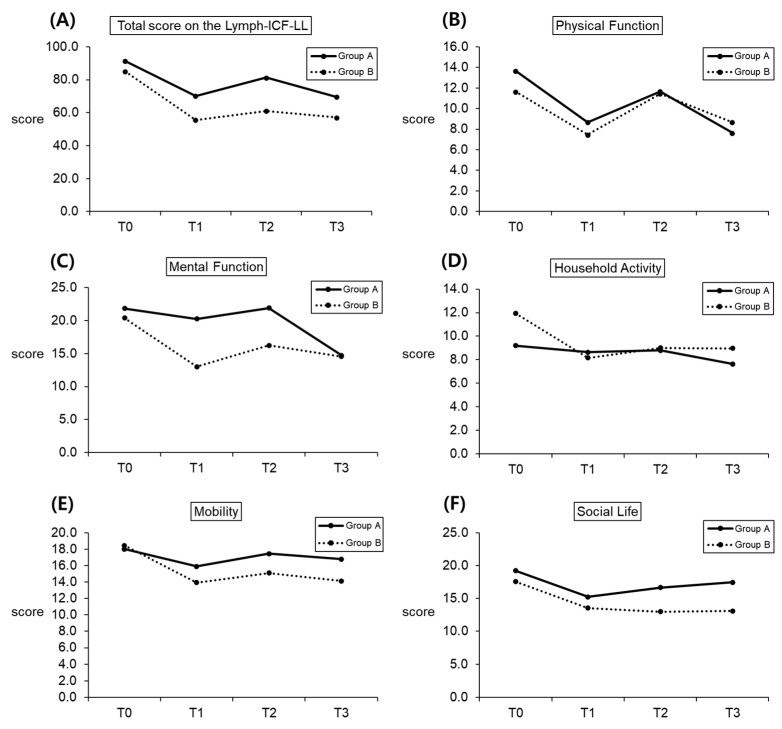
Serial change in subgroups for the total ICF score and scores on the five domains: (**A**) total score on the Lymph-ICF-LL; (**B**) physical function (**C**) mental function, (**D**) household activity, (**E**) mobility, and (**F**) social life. T0: baseline; T1: post-intervention; T2: one-month follow-up; and T3: two-month follow-up.

A comparison of time-dependent changes in the ICF domain scores between the subgroups is shown in Table 5 and Figure 2A–F. Both groups presented a decline in total ICF scores and all domain scores after the four-week intervention compared with baseline, with no statistically significant differences between the two groups. At the one-month follow-up, all scores increased compared to the post-intervention results in both groups without any significant inter-group differences.

### 3.6. Satisfaction Scores

**Figure 3:**
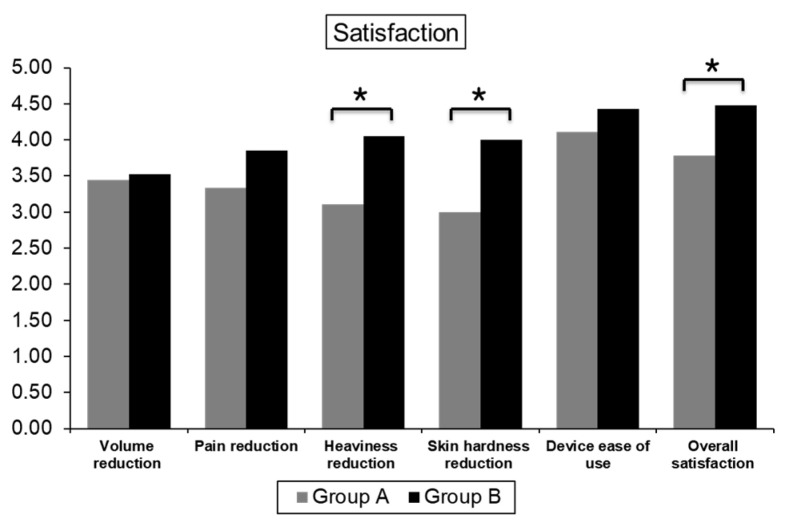
Comparison of satisfaction score between subgroups. * *p* value < 0.05.

The mean overall satisfaction score, rated on a 5-point scale (1: not at all, 5: very much) was 4.27 ± 0.77. When the participants were grouped according to their compliance with routine compression bandaging during the intervention period, the overall satisfaction score was significantly higher in group B (good compliance with routine compression bandaging) than in group A (*p* = 0.031). The two questionnaire items showed significant differences between the groups in terms of reduction in heaviness and skin hardness. Group B reported significantly more relief from heaviness and skin hardness on the affected limb than group A after four weeks of home-pump application. For volume reduction, pain, and ease of use, group B presented slightly higher scores than group A, but the differences were not statistically significant (Table 5 and Figure 3).

## 4. Discussion

The main purpose of this single-arm study was to evaluate the efficacy in inter-limb difference ratio, QOL, and satisfaction of a home-based IPC device with an MLD-mimicking program for the treatment of gynecologic cancer patients with secondary unilateral leg lymphedema during the maintenance phase. A major strength of this study is the application of a new-generation IPC device that mimics MLD and follow-up data after the four-week intervention period. There was no statistically significant decrease in the V*_ratio_* of the proximal, distal, and whole limbs, and this appeared to be maintained throughout the study period. Importantly, QOL was significantly improved in all domains, and the participants expressed high satisfaction with the intervention. To our knowledge, only one clinical trial has reported the use of this new-generation IPC device. In addition, the outcome measure from the post-intervention period may provide clues to the remaining effect of IPC application.

In our study, there were no significant time-dependent interactions in the V*_ratio_* of the proximal, distal, and whole limbs. However, participants who used the home-based IPC device and continued using short-stretch bandages (group B) showed a significantly greater decline in the V*_ratio_* after the intervention than those who followed the home-based IPC device protocol without maintaining their use of short-stretch bandages (group A). This difference increased significantly in the distal part of the limb after one month without access to the home-based IPC device. This primary outcome implies that the IPC device may be used for volume reduction during the maintenance phase and may help boost compliance among chronic patients.

In terms of QOL, the total score and all four domain scores improved significantly after the intervention, regardless of compliance with the short stretch bandage. Although this improvement decreased slightly at the one-month follow-up, sustainable improvement was observed at both one and two months post-intervention, regardless of compliance with the short-stretch bandage routine. Thus, the home-based IPC device played a positive role in improving the QOL of patients with CLL. During the intervention, many participants felt more confident during daily living and reported active participation in social and occupational activities that were previously impossible, such as taking a backpacking trip and playing table tennis.

Our findings are consistent with those of previous studies [18]. Only one pilot randomized controlled trial (RCT) in 20 patients with lower-limb lymphedema diagnosed at stage 2 investigated the feasibility of a home-based IPC device over six months. The home-based IPC device used in a previous study first applied compression from the proximal aspect to the distal aspect with a pressure of 40 mmHg for 35-min cycles (for safety purposes). In contrast, our study applied 40–60 mmHg pressure in 30 min of the first mode (from proximal to distal aspect), and then 80–100 mmHg pressure in 30 min of the second mode (from distal to proximal aspect). The recommended frequency is twice daily. In both studies, the mean changes in the affected limb volume or inter-limb volume ratio were not significantly different within or between the groups over time. In the pilot RCT trial, the mean QOL scores in both groups decreased slightly, but they did not examine the statistical significance between groups and within each group over time. As the QOL measurement tools were different, the results of the two studies may not be consistent. Both studies demonstrated the good usability of the home-based IPC device.

Lymphedema is a chronic disease that requires delicate treatment. Improperly treated lymphedema causes physical discomfort and functional impairment. Compared with uterine cancer survivors without leg lymphedema, survivors with leg lymphedema were more likely to present poor physical function [24]. In addition to physical problems, patients with chronic lymphedema suffer from negative psychological effects, such as negative self-identity and emotional disturbance, and negative social effects that range from a financial burden to a non-supportive work environment [25].

Despite the clinical efficacy of CDT, the time required and its high cost are obstacles for chronic lymphedema patients [26]. Home-based IPC devices have been highlighted as a potential substitute for CDT. In a cancer-related lymphedema database study, IPC use was associated with reduced hospitalization, outpatient hospital visits, cellulitis diagnoses, and physical therapy use, resulting in decreased healthcare costs [27]. However, for various reasons, such as the high cost of initial models, contradictory clinical outcomes, safety concerns about home-based medical devices, and neglect of the Korean national medical insurance system, home-based IPC devices have not been established as an adjuvant treatment option. In this study, we revealed that a home-based IPC device with an MLD-mimicking mode could be safely used as an adjuvant home therapy alongside conservative compression therapy in well-trained patients with stage 3 chronic leg lymphedema.

Home-based IPC devices have potential advantages. In particular, patients can save transportation costs and time by using IPC with the MLD function and performing routine self-care in comparison to physiotherapy in person. In addition, in patients who have difficulty visiting specialized hospitals, IPC with an MLD function can have a role in reducing the barriers to health services. Moreover, many participants reported that the use of home-based IPC devices made them feel psychologically safe during participation in social, occupational, and sports activities, as well as daily living. Although there was no substantial change in volume, the patients experienced improvement in subjective symptoms or signs. In summary, the use of home IPC devices is meaningful in reducing the burden of self-management by reducing symptoms, promoting activities of daily living, and saving time and money in patients who spend considerable time and effort to maintain lower extremity volume.

However, some limitations of our study must be addressed. First, the clinical significance of the results was limited owing to the single-group design. Although there are many threats to the internal validity of the one-group pre-test-post-test designs, we used this design. The main reason for this is the time restrictions and funding necessary to enroll the case-control participants. Additional explanations may be of benefit to a single-group design that provides rapid results for treatment effectiveness within a limited timeframe. No study has confirmed the effectiveness of the home IPC equipment used in this study. In addition, patients with unilateral lymphedema were chosen because they were more commonly accessible; therefore, it can help ensure subject homogeneity and minimize measurement bias in the primary outcome (ratio calculated based on the volume difference between the affected and non-affected sides). Second, the total number of participants was too small to support further correlation analysis. Third, although compliance with the intervention device was 100% in all participants, the intervention period of four weeks was shorter than that in other studies on IPC devices [1,28]. Fourth, the relatively short follow-up period limited the interpretation of our results. However, because home-based IPC devices are expensive, it is necessary to determine their short-term effects before their long-term effects on chronic lymphedema can be considered relevant. Therefore, we chose short intervention and follow-up periods for this study. Lastly, the mean duration of lymphedema (15.46 ± 7.57 years) of the participants may have influenced the result. Further studies with a case-control design, larger samples, longer intervention periods, and long-term follow-up results are needed. Additionally, compliance with home-based IPC programs and self-routine care was dependent on patient-reported questionnaires. Because it can be a possible source of bias within the study, future studies need to consider a quantitative method, for example, remote monitoring.

## 5. Conclusions

In conclusion, this single-arm study identified that a home-based IPC device with an MLD-mimicking program is a useful option for maintaining the volume of limbs and improving the QOL of patients with stage 3 chronic leg lymphedema during the maintenance phase. Moreover, it can be significant in reducing the burden of self-management by relieving symptoms, promoting the activities of daily living, and saving the time and money of patients who invest considerable effort in maintaining lower extremity volumes. In the future, further larger scale, randomized control trials are required to demonstrate the definitive efficacy of the additional use of a home-based IPC device operating on a mixed MLD process (proximal to distal compression) and common mode (distal to proximal compression) design.

## Figures and Tables

**Table 1:** General characteristic of participants.

Parameters		Participants (*n* = 30)	Group A (*n* = 9)	Group B (*n* = 21)	*p*-Value
Sex (female (%))		0/30 (100%)	0/9 (100%)	0/21 (100%)	
Age (years, mean ± SD)		56.47 ± 11.33	62.22 ± 6.02	54.00 ± 12.27	0.068
Height (cm, mean ± SD)		159.95 ± 5.97	157.89 ± 6.19	160.83 ± 5.79	0.244
Initial BMI (kg/m^2^, mean ± SD)		23.87 ± 3.33	24.39 ± 2.67	23.80 ± 3.02	0.158
Onset of lymphedema after surgery (years, mean ± SD)		3.18 ± 2.58	3.23 ± 2.04	3.16 ± 3.21	0.782
Mean duration of lymphedema (years, mean ± SD)		15.46 ± 7.57	14.42 ± 7.92	15.91 ± 5.84	0.323
Affected side, N (%)	Right	17 (56.7)	4 (44.4)	13 (61.9)	0.376
	Left	13 (43.3)	5 (55.6)	8 (38.1)	
Chemotherapy, N (%)		11 (36.7)	3 (33.3)	8 (38.1)	0.804
Radiotherapy, N (%)		11 (36.7)	5 (55.6)	6 (28.6)	0.160
Cancer type, N (%)					
Ovarian cancer		6 (20.0)	2 (22.2)	4 (19.0)	0.842
Cervical cancer		16 (53.3)	4 (44.4)	12 (57.1)	0.523
Fallopian tube		2 (6.7)	0 (0)	2 (9.5)	0.338
Endometrial cancer		6 (20.0)	3 (33.3)	3 (14.3)	0.232

SD = standard deviation; BMI = body mass index.

**Table 2:** Volume difference ratio of the distal, proximal, and whole limb of the affected side at baseline (T0), post-intervention (T1), one-month follow-up (T2), and two-month follow-up (T3).

Outcome	T0 (*n* = 30)	T1 (*n* = 30)	T2 (*n* = 30)	T3 (*n* = 30)	χ^2^ (df)	*p*-Value
	Mean (SD)					
Distal limb volume difference ratio	22.21 (17.67)	22.07 (17.42)	21.97 (18.07)	23.19 (17.92)	2.40 (3)	0.494
Proximal limb volume difference ratio	23.86 (11.27)	23.41 (11.49)	23.63 (12.05)	25.67 (13.43)	6.85 (3)	0.077
Whole limb volume difference ratio	23.15 (12.92)	22.83 (12.77)	23.03 (13.09)	24.65 (13.98)	4.92 (3)	0.178

SD = standard deviation.

**Table 3:** Comparison of the lower-limb volume ratio between subgroups.

Outcome	Group A (*n* = 9)		Group B (*n* = 21)		Mean Difference (95% CI)		*p*-Value
	Mean (SD)						
Distal limb volume difference ratio							
△T0–T1	−2.60	(4.21)	5.60	(5.28)	−8.20	(−11.90, −4.49)	<0.001 *
△T1–T2	1.37	(3.76)	−3.54	(5.42)	4.91	(1.40, 8.42)	0.008 *
△T2–T3	0.75	(4.23)	2.32	(7.86)	−1.57	(−7.76, 4.61)	0.584
Proximal limb volume difference ratio							
△T0–T1	−1.91	(3.87)	2.97	(4.53)	−4.88	(−8.20, −1.56)	0.006 *
△T1–T2	0.79	(2.61)	−1.12	(4.55)	1.90	(−0.78, 4.58)	0.157
△T2–T3	1.37	(5.09)	3.62	(5.94)	−2.26	(−6.62, 2.11)	0.299
Whole limb volume difference ratio							
△T0–T1	−2.22	(2.96)	4.10	(4.01)	−6.33	(−9.02, −3.64)	0.000 *
△T1–T2	1.20	(2.47)	−2.13	(4.52)	3.33	(0.73, 5.94)	0.014
△T2–T3	0.97	(2.47)	3.17	(6.55)	−2.20	(−6.23, 1.82)	0.272

SD = standard deviation; CI = confidence interval; T0 = baseline; T1 = post-intervention; T2 = one-month follow up; T3 = two-month follow up. * *p* value < 0.008 (0.05/6).

**Table 4:** ICF scores (total score and five domain scores) at baseline (T0), post-intervention (T1), one-month follow-up (T2), and two-month follow-up (T3).

ICF Domain	RMANOVA (or Friedman Test)					Pairwise Comparisons (or Wilcoxon Signed-Rank Test)					
	Mean (SD)										
	T0	T1	T2	T3	*p* Value	T0 vs. T1	T0 vs. T2	T0 vs. T3	T1 vs. T2	T1 vs. T3	T2 vs. T3
Total score	92.57 (37.34)	57.90 (29.33)	65.49 (31.45)	61.07 (34.20)	<0.001 *	<0.001 *	<0.001 *	<0.001 *	0.048 *	0.426	0.225
Physical function	12.03 (7.71)	6.33 (5.47)	9.33 (7.77)	8.67 (8.33)	0.013	0.001 *	0.164	0.040 *	0.009 *	0.141	0.638
Mental function	28.27 (16.19)	16.70 (16.14)	18.73 (14.15)	16.80 (14.30)	<0.001 *	<0.001 *	0.004 *	<0.001 *	0.204	0.809	0.313
Household activity	10.10 (7.12)	5.27 (3.90)	5.63 (4.08)	5.47 (4.84)	0.005 *	0.001 *	0.003 *	0.001 *	0.319	0.508	0.863
Mobility	22.90 (13.04)	15.37 (9.56)	17.17 (10.42)	15.63 (9.61)	0.001 *	0.004 *	0.008 *	0.002 *	0.262	0.866	0.226
Social life	19.27 (6.75)	13.93 (7.20)	14.63 (7.35)	14.50 (8.79)	0.003 *	0.005 *	0.004 *	0.014 *	0.436	0.569	0.906

RMANOVA = repeated-measures analysis of variance; SD = standard deviation. T0 = baseline; T1 = post-intervention; T2 = one-month follow up; T3 = two-month follow up. * *p* value < 0.05.

**Table 5:** Comparison of ICF domain scores and satisfaction between subgroups.

Outcome	Group A (*n* = 9)		Group B (*n* = 21)		*p*-Value
	Mean (SD)				
**Quality of life**					
Total ICF score					
△T0–T1	21.22	(32.90)	40.43	(42.59)	0.239
△T1–T2	−11.22	(23.76)	−5.86	(18.22)	0.505
△T2–T3	11.67	(24.25)	0.95	(14.85)	0.245
Physical function					
△T0–T1	5.00	(8.32)	5.57	(7.12)	0.849
△T1–T2	−3.00	(5.00)	−2.57	(4.91)	0.829
△T2–T3	2.33	(7.70)	−0.05	(6.02)	0.369
Mental function					
△T0–T1	4.33	(13.67)	14.67	(15.14)	0.135
△T1–T2	−5.00	(9.11)	−0.76	(12.54)	0.319
△T2–T3	9.89	(11.17)	−1.48	(10.27)	0.014
Household activity					
△T0–T1	3.11	(5.11)	5.57	(7.71)	0.468
△T1–T2	−0.22	(5.47)	−0.43	(2.42)	0.475
△T2–T3	−0.44	(5.92)	0.43	(3.96)	0.964
Mobility					
△T0–T1	4.78	(7.71)	8.71	(14.96)	0.541
△T1–T2	−1.56	(10.51)	−1.90	(7.96)	0.633
△T2–T3	0.67	(6.08)	1.90	(7.18)	0.510
Social life					
△T0–T1	4.00	(9.72)	5.90	(9.57)	0.587
△T1–T2	−1.44	(4.13)	−0.19	(3.88)	0.663
△T2–T3	−0.78	(6.96)	0.14	(5.87)	0.453
**Satisfaction**					
Overall satisfaction	3.78	(0.83)	4.48	(0.68)	0.031 *
Volume reduction	3.44	(0.88)	3.53	(1.17)	0.760
Pain reduction	3.33	(0.71)	3.86	(1.01)	0.162
Heaviness reduction	3.11	(0.78)	4.05	(0.86)	0.012 *
Skin hardness reduction	3.00	(0.87)	4.00	(0.77)	0.007 *
Device ease of use	4.11	(1.05)	4.43	(0.68)	0.516

SD = standard deviation; T0 = baseline; T1 = post-intervention; T2 = one-month follow up; T3 = two-month follow up. The adjusted significance level was set at *p* = 0.008 (quality of life). * *p* value < 0.05 (satisfaction).

## Data Availability

The data are available to academic researchers upon request.
